# Rhizobacteria Increase the Adaptation Potential of Potato Microclones under Aeroponic Conditions

**DOI:** 10.3390/microorganisms11071866

**Published:** 2023-07-24

**Authors:** Oksana V. Tkachenko, Nina V. Evseeva, Kristina Y. Kargapolova, Alena Y. Denisova, Natalia N. Pozdnyakova, Artem A. Kulikov, Gennady L. Burygin

**Affiliations:** 1 Department of Plant Breeding, Selection, and Genetics, Faculty of Agronomy, Saratov State University of Genetics, Biotechnology and Engineering named after N.I. Vavilov, 410012 Saratov, Russia; kinaschchri@gmail.com (K.Y.K.); alena.denisova1408@yandex.ru (A.Y.D.); artyomka.net@mail.ru (A.A.K.); burygingl@gmail.com (G.L.B.); 2 Institute of Biochemistry and Physiology of Plants and Microorganisms, Saratov Scientific Centre of the Russian Academy of Sciences (IBPPM RAS), 410049 Saratov, Russia; evseeva_n@ibppm.ru (N.V.E.); pozdnyakova_n@ibppm.ru (N.N.P.); 3 Department of Organic and Bioorganic Chemistry, Institute of Chemistry, Saratov State University, 410012 Saratov, Russia

**Keywords:** adaptation ex vitro, antioxidant enzymes, plant-growth-promoting rhizobacteria, *Solanum tuberosum* L.

## Abstract

Adaptation ex vitro is strongly stressful for microplants. Plant-growth-promoting rhizobacteria (PGPR) help to increase the adaptation potential of microplants transplanted from test tubes into the natural environment. We investigated the mechanisms of antioxidant protection of PGPR-inoculated potato microclones adapting to ex vitro growth in an aeroponic system. Potato (*Solanum tuberosum* L. cv. Nevsky) microplants were inoculated in vitro with the bacteria *Azospirillum baldaniorum* Sp245 and *Ochrobactrum cytisi* IPA7.2. On days 1 and 7 of plant growth ex vitro, catalase and peroxidase activities in the leaves of inoculated plants were 1.5-fold higher than they were in non-inoculated plants. The activity of ascorbate peroxidase was reduced in both in vitro and ex vitro treatments, and this reduction was accompanied by a decrease in the leaf content of hydrogen peroxide and malondialdehyde. As a result, inoculation contributed to the regulation of the plant pro/antioxidant system, lowering the oxidative stress and leading to better plant survival ex vitro. This was evidenced by the higher values of measured morphological and physiological variables of the inoculated plants, as compared with the values in the control treatment. Thus, we have shown some PGPR-mediated mechanisms of potato plant protection from adverse environmental factors under aeroponic conditions.

## 1. Introduction

Clonal micropropagation in vitro produces the required number of healthy microclones of vegetatively propagated plants, including potato. In recent years, aeroponics [1] has been commonly used to improve the quality and quantity of minitubers produced from potato microplants. This technique can increase the reproduction coefficient and accelerate the production of healthy seeds. In addition, substrate-free cultivation can be used to model the physiology of microplant adaptation ex vitro [2].

The inoculation of plants with beneficial microorganisms [3] is of high interest for potato production and seed breeding. The adaptation potential of microclones can be improved considerably by establishing active associations between microplants and plant-growth-promoting rhizobacteria (PGPR). Such associations increase the growth rate of microclones and speed up the induction of plant defense against abiotic and biotic environmental stressors [4]. Previously, we have shown that single-strain and two-strain inoculations with rhizospheric strains *Azospirillum baldaniorum* Sp245 and *Ochrobactrum cytisi* IPA7.2 benefit the growth and development of potato under in vitro conditions [5,6] and in greenhouses [7]. In addition, we have established that *A. baldaniorum* Sp245 promotes potato productivity under field and aeroponic conditions [8,9]. Positive effects of plant-growth-promoting microorganisms in soil-free systems have also been shown with other plant species, including banana, strawberry, lettuce, tomato, soybean, and wheat [10]. In leaf lettuce, salt stress was strongly alleviated by the addition of a bacterial biostimulant to a hydroponic nutrient solution [11].

Transfer to ex vitro growth conditions is stressful for microplants. Stress leads to an increase in the production of reactive oxygen species (ROS). However, in response to the negative effects of ROS, cells produce antioxidant enzymes and organic compounds that are able to stop potential damage [12].The main physiological and biochemical indices examined in stressed plants are the content of MDA as the end product of lipid peroxidation; the content of photosynthetic pigments; the activity of antioxidant enzymes (e.g., peroxidase, ascorbate peroxidase and catalase), which detoxify hydrogen peroxide, a major ROS component, and superoxide dismutase, which catalyzes the dismutation of the superoxide radical into oxygen and hydrogen peroxide [13]. Although the functioning of antioxidant systems in microplants acclimating to planting conditions has been examined [14], the use of plant bacterization in aeroponics is poorly understood. Rhizobacteria promote the yield of minitubers [15] and improve the resistance of potato to pathogens [16], but almost nothing is known about their effect on the antioxidant protection mechanisms of microplants adapting to aeroponic growth.

Here we study the mechanisms of antioxidant protection in PGPR-inoculated potato microclones adapting to growth in an aeroponic system (ex vitro). In plants inoculated with two strains of rhizobacteria in vitro and then planted in aeroponic conditions, the complex of morphological parameters were evaluated, such as the length and number of roots, the length of shoots, the number of nodes, the mass of shoots and roots, the number and size of stomata on leaves, as well as the productivity of mini-tubers. In addition, biochemical parameters characterizing the dynamics of the state of the pro/antioxidant system were determined, including the content of malondialdehyde (MDA), hydrogen peroxide and antioxidant enzymes (catalase, peroxidase, and ascorbate peroxidase) in the leaves. The data obtained were necessary to assess the magnitude of stress during the transition from favorable conditions in vitro to the more difficult conditions of aeroponics and the role of rhizobacteria in stress regulation.

## 2. Materials and Methods

### 2.1. Plant Material

Potato (*Solanum tuberosum* L. cv. Nevsky) microplants were obtained from the in vitro potato microclone collection of the Department of Plant Breeding, Selection, and Genetics of the Faculty of Agronomy at Saratov State University of Genetics, Biotechnology and Engineering named after N.I. Vavilov (Vavilov University). The microplants had been produced by isolation of apical meristems. The middle–early Nevsky cultivar (ZAO Vsevolozhskaya selektsionnaya stantsiya, Russian Federation) is high yielding and adapted to different soils and climates [17].

### 2.2. Characterization of Bacteria

Bacteria were obtained from the Collection of Rhizosphere Microorganisms, Institute of Biochemistry and Physiology of Plants and Microorganisms, Russian Academy of Sciences (IBPPM RAS) (http://collection.ibppm.ru (accessed on 11 July 2023)). *Azospirillum baldaniorum* Sp245 (IBPPM 219) [18]. A diazotroph isolated from surface-disinfected wheat roots in Brazil [19], is a facultative endophyte [20] that promotes the growth of a wide range of cultivated plants [21]. *Ochrobactrum cytisi* IPA7.2 (IBPPM 544) was isolated by us from the rhizosphere of the Nevsky potato cultivar in Saratov Region (Russian Federation). It colonizes plant roots and promotes the growth of potato microplants [5].

### 2.3. Experimental Plan

Microplants were cut into cuttings, each of which contained one axillary bud, and the cuttings were placed in test tubes filled with a nutrient medium. Shoot cultures were grown for 30 days in a liquid hormone-free Murashige–Skoog medium [22]. The cultivation temperature was maintained at 24 °C, the air humidity was 60%, the illumination intensity was 60 μM m^−2^ s^−1^ and the day duration was 16 h. Explants were inoculated with a suspension of *A. baldaniorum* Sp245 on day 0 of cultivation and additionally with a suspension of *O. cytisi* IPA7.2 after 14 days of growth. Previously, we have already established the positive effect of co-inoculation with two strains of *A. baldaniorum* Sp245 and *O. cytisi* IPA7.2 for the adaptation of potato microplants in greenhouse conditions [7], as well as with one strain of *A. baldaniorum* Sp245 in aeroponics conditions [9]. Therefore, in this study, both strains were used for an experiment in aeroponics. In addition, earlier we found that optimal conditions for inoculation of potato microplants in in vitro culture for the *A. baldaniorum* Sp245 strain were 0 days of cultivation [6], and for the *O. cytisi* IPA7.2 strain were 14 days of cultivation [5]. Bacterial cultures had been grown in a liquid malate–salt medium for 18 h at a temperature of 35 °C and had been prepared for inoculation as described earlier [6]. The bacterial cell concentration in the Murashige–Skoog medium after inoculation was 10^6^ cells mL^−1^ for both strains, and non-inoculated plants were used as the control. Thus, the duration of in vitro co-cultivation of the microplants was 30 days with *A. baldaniorum* Sp245 and 16 days with *O. cytisi* IPA7.2.

**Figure 1:**
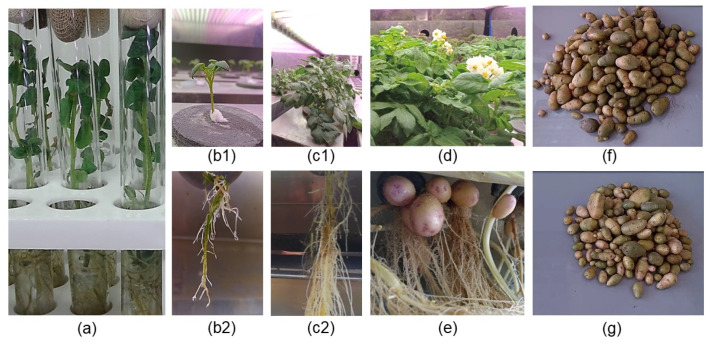
Adaptation of potato microplants to aeroponic growth: (**a**) 30-day-old microplants in vitro; (**b**) third day of aeroponic growth ((**b1**) shoot, (**b2**) roots); (**c**) fourteenth day of aeroponic growth ((**c1**) shoot, (**c2**) roots); (**d**) fiftieth day of aeroponic growth; (**e**) potato minitubers in the aeroponic system; (**f**) yield of tubers of 10 plants in the experiment; (**g**) yield of tubers of 10 plants in the control.

Thirty-day-old microplants were planted in an aeroponic system (Figure 1). The plants were grown under a 16-h day and 8-h night photoperiod at a daytime temperature of 25 °C and a night-time temperature of 20 °C. The composition of the plant growth nutrient solution was described by Tkachenko et al. [9]. After 2 weeks of growth, the concentration of the main nutrient solution was doubled. In the first 2 weeks of growth, the period for which the solution was sprayed was 2 min and the period for which it stayed in the air was 5 min; later, the former period was 5 min and the latter period was 15 min. The adaptation in the aeroponic system lasted 3 weeks. After that, the plants were grown further to obtain a yield of minitubers. During tuber formation, the daylight length was 12 h and the temperature was 20 °C during the day and 18 °C during the night. The total duration of aeroponic growth was 4 months.

### 2.4. Morphometric Analysis

After 30 days of in vitro cultivation, the growth variables of the microplants were measured, including lengths and number of roots, length of shoot, number of nodes, and fresh and dry weight of roots and shoots.

On day 21 of aeroponic growth, lengths of roots and shoots, number of leaves, leaf area per plant, average area per leaf, and fresh and dry weights of shoots and roots were measured. In each experimental treatment, 10 plants were examined.

### 2.5. Analysis of Stomata

Stomata on the leaves of the central plant part were analyzed on days 0, 10, and 20 of aeroponic growth. Epidermal tissue was removed from the abaxial leaf surface, and the number of stomata per 0.1 mm^2^, the width of the stomatal pore, and the area of stomata were determined. Slides were observed under an Olympus CX23 optical microscope (Olympus, Tokyo, Japan) equipped with a digital camera. In each experimental treatment, density measurements were made on the basis of 10 images, whereas the average values of the stomatal complex size were based on 30 measurements (*n* = 30).

### 2.6. Measurement of Photosynthetic Pigments

The content of photosynthetic pigments (chlorophylls *a* [C*a*] and *b* [C*b*] and carotenoids [C*x + c*]) in the leaves was measured according to Wellburn [23] and to Hiscox and Israelstam [24]. In each treatment, we firstly used normally formed leaves from the apical buds of four plants. Each analyzed leaf was weighed and placed in dimethyl sulfoxide at a ratio of 50 mg to 5 mL of dimethyl sulfoxide. The samples were incubated at 65 °C for 120 min. The extracts from each of the samples were analyzed in three analytical replicates (*n* = 12). The absorbance was measured at 480 nm (*A*_480_), 649 nm (*A*_649_), and 665 nm (*A*_665_) on a Specord 250 spectrophotometer (Analytik Jena GmbH+Co., Jena, Germany). The content of photosynthetic pigments was calculated according to the following formulae: C*a* = 12.19 × *A*_665_ − 3.45 × *A*_649_; C*b* = 21.99 × *A*_649_ − 5.32 × *A*_665_; C*x + c* = (1000 × *A*_480_ − 2.14 × C*a* − 70.16 × C*b*)/220.

### 2.7. Measurement of Malondialdehyde (MDA)

Malondialdehyde was determined by photometric method. The level of coloration during the formation of a complex with thiobarbituric acid during heating was evaluated [25]. To this end, 100 mg of leaf tissue was homogenized in 1.5 mL of 20% trichloroacetic acid and the homogenate was centrifuged (10,000× *g*, 4 °C, 15 min). Then, 0.3 mL of the supernatant liquid was mixed with 1.2 mL of 20% trichloroacetic acid containing 0.5% thiobarbituric acid. The mixture was heated in a water bath (95 °C, 30 min). The reaction mixture was then recentrifuged (10,000× *g*, 4 °C, 5 min), and the absorbance of the supernatant liquid was measured at 532 and 600 nm. The concentration of MDA was calculated from its extinction coefficient (156 mM^−1^ cm^−1^). Samples were examined in four analytical replicates, with three samples in each replicate (*n* = 12).

### 2.8. Measurement of Antioxidant Enzyme Activities

Enzymes were extracted from leaves according to Dubrovskaya et al. [26]. Peroxidase activity was measured by the oxidation of 2,7-diaminofluorene at 600 nm [27]. Catalase and ascorbate peroxidase activities were measured by the decomposition of hydrogen peroxide and the oxidation of ascorbic acid at 240 nm and 290 nm, respectively [28]. Enzyme activity was determined on an Evolution™ 60S UV-Visible spectrophotometer (Thermo Scientific, Waltham, MA, USA). One unit of enzyme activity is defined as the amount required to catalyze the formation of 1 µM of the product or the disappearance of 1 µM of the substrate per min. Protein was measured according to Bradford [29]. Samples were examined in four analytical replicates, with three samples in each replicate (*n* = 12).

### 2.9. Measurement of Hydrogen Peroxide

The leaf content of hydrogen peroxide was measured by the method of Yarullina et al. [30], with modifications. Leaves were homogenized at 4 °C in a mortar in 25 mM of phosphate buffer (pH 6.2; leaf-to-buffer ratio (*m*/*v*), 1:3) and were centrifuged at 10,000× *g* and 4 °C for 15 min. Hydrogen peroxide was measured in the supernatant liquid by using xylenol orange. The resulting mixture was kept at a temperature of 20 °C for 40 min and then the absorption spectrum was measured at 560 nm (Specord 250, Analytik, Jena, Germany). Samples were examined in four analytical replicates, with three samples in each replicate (*n* = 12).

### 2.10. Detection of Bacteria in Plant Roots

On days 0, 5, 7, 14, and 21 of aeroponic growth, bacteria were detected in plant roots by enzyme-linked immunosorbent assay (ELISA). The 2-cm terminal parts of three roots were homogenized and incubated at 100 °C for 10 min to inactivate peroxidase [31]. The specific antibodies were those directed to the O antigens of *A. baldaniorum* Sp245 [32] and *O. cytisi* IPA7.2 [5]. The number of bacteria in the root homogenates was calculated by the absorbance at 492 nm of the solutions after the enzymatic reaction. In each experimental treatment, root homogenates were analyzed in four analytical replicates (*n* = 4). A calibration curve was plotted by using bacterial suspensions with known cell numbers.

### 2.11. Yield Analysis

As soon as tubers formed, they were collected at 10 day intervals. Minitubers of 1.5 cm size or greater were harvested and stored in a refrigerator at 15 °C. After the end of growth, the number of minitubers on the plant, their average mass and diameter were counted to analyze the yield.

### 2.12. Statistical Analysis

Data were processed by one-way and two-way ANOVA (factor A: type of experiment (control and treated plants); factor B: growth period (days)). To compare the averages for the experimental treatments, the least significant difference (LSD) and the statistical significances of the difference between means by the Duncan’s test were determined at a significance level of 95% (*p ≤* 0.05). The ranking according to the Duncan’s test is indicated in the tables and diagrams by different Latin letters. Two independent experiments were conducted.

## 3. Results

### 3.1. Plant–Bacterial Associations In Vitro

Co-inoculation with *A. baldaniorum* Sp245 and *O. cytisi* IPA7.2 promoted the growth of potato microclones. The experimental plants had greater shoot lengths (+7.4%), fresh and dry shoot weights (+29 and +44%, respectively), and fresh and dry root weights (+21.6 and +80%, respectively) (Table 1). In addition, the ratio between root dry weight and root fresh weight in the treated plants was 1.5 times higher than that in the control plants. In the inoculated plants, the number of roots increased significantly (+4.2%), whereas their length decreased (−11.7%). The number of shoot nodes did not differ between control and treated microplants. These data confirm our preliminary results that both strains promote potato growth and development of potato in vitro and ex vitro [7].

**Figure 2:**
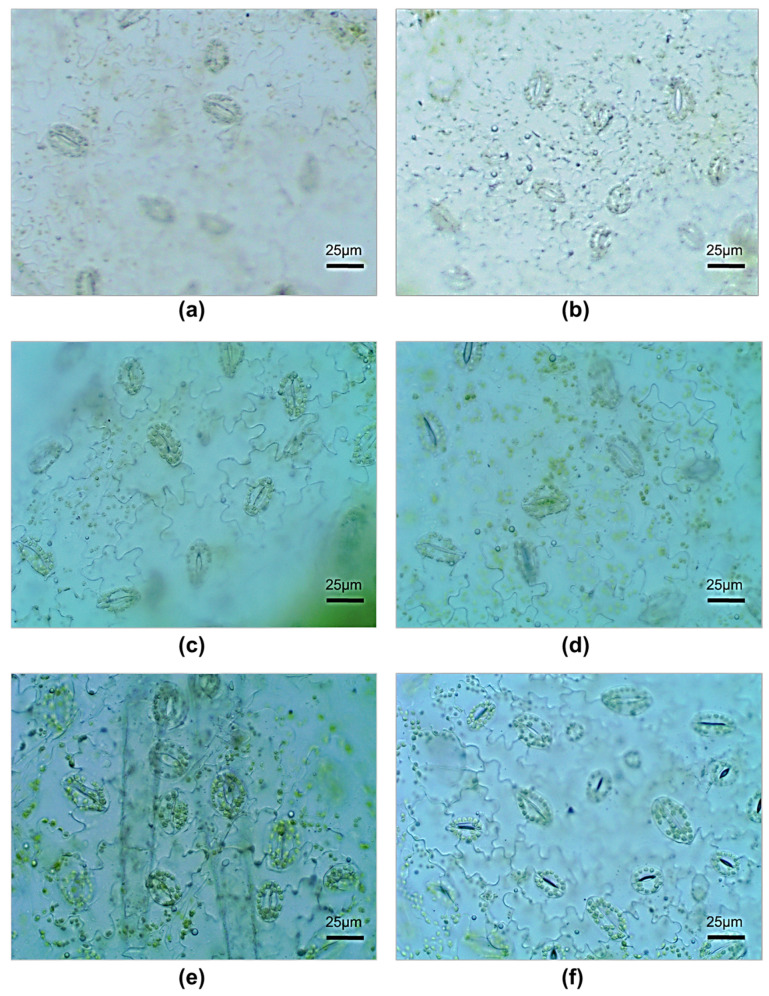
Stomata of potato microplants: (**a**) 30-day-old microplants in vitro (control); (**b**) 30-day-old microplants in vitro (treated plants); (**c**) day 10 of aeroponic growth (control); (**d**) day 10 of aeroponic growth (treated plants); (**e**) day 20 of aeroponic growth (control); (**f**) day 20 of aeroponic growth (treated plants).

**Figure 3:**
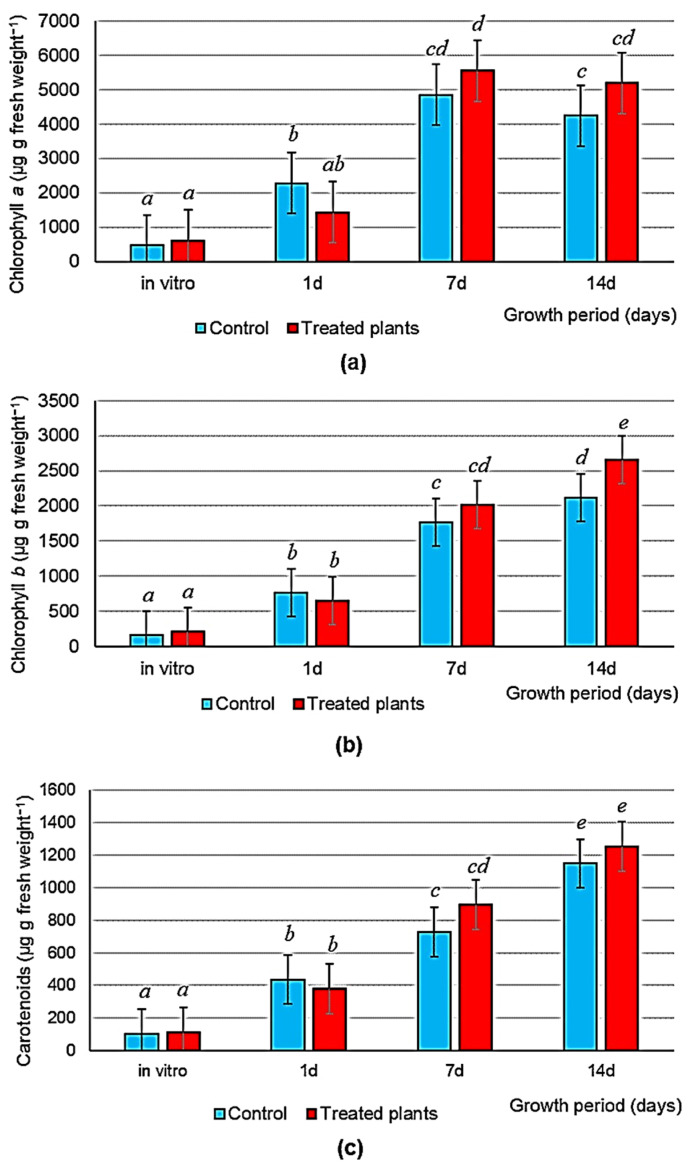
The content of photosynthetic pigments in leaves of non-inoculated plants (control) and inoculated with *A. baldaniorum* Sp245 and *O. cytisi* IPA7.2 (treated plants) in vitro and on days 1, 7 and 14 of aeroponic growth: (**a**) the content of chlorophyll *a*; (**b**) the content of chlorophyll *b*; (**c**) the content of carotenoids. Data were processed by one-way and two-way ANOVA (factor A: type of experiment (control and treatment); factor B: growth period (days)). The bars represent LSD for experience options. Latin letters indicate differences between treatments according to the results of the Duncan’s test at *p* ≤ 0.05. *n* = 12.

In vitro, the inoculated plants did not differ from the control ones in the size and number of stomata (Table 2, Figure 2) or in the content of photosynthetic pigments (Figure 3).

**Figure 4:**
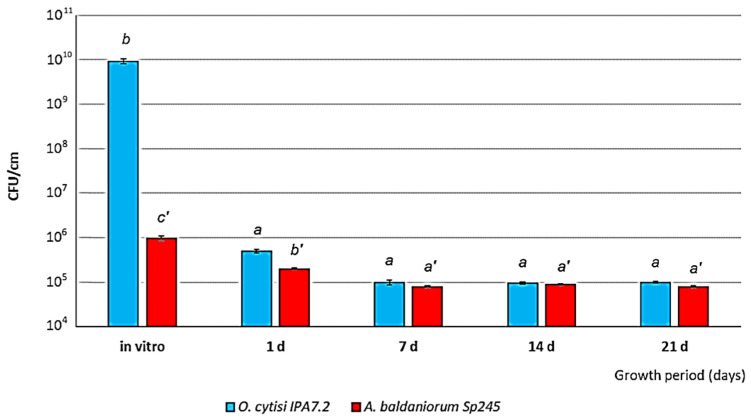
Numbers of cells of *A. baldaniorum* Sp245 and *O. cytisi* IPA7.2 in the roots of plants in vitro and on days 1, 7, 14, and 21 of aeroponic growth. Data were processed by one-way ANOVA for each strain. The bars represent LSD for experience options. Latin letters (*a*, *b*, *a*′, *b*′ and *c*′) indicate differences between treatments according to the results of the Duncan’s test at *p* ≤ 0.05. *n* = 4.

On day 30 of plant growth in vitro, both bacterial strains were present in large numbers in the roots of the microplants as confirmed by enzyme-linked immunosorbent assay (ELISA) (Figure 4).

**Figure 5:**
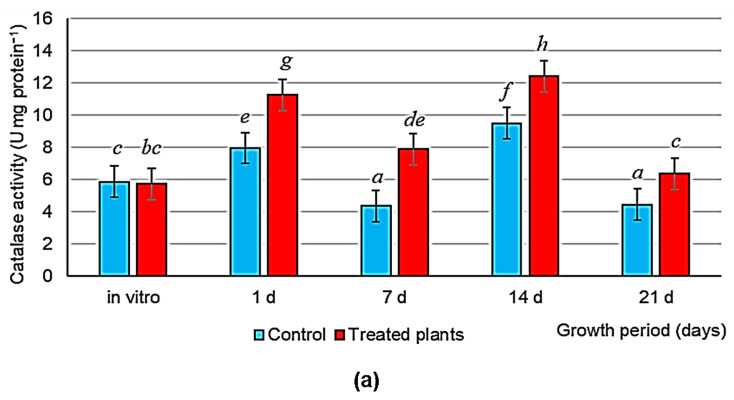

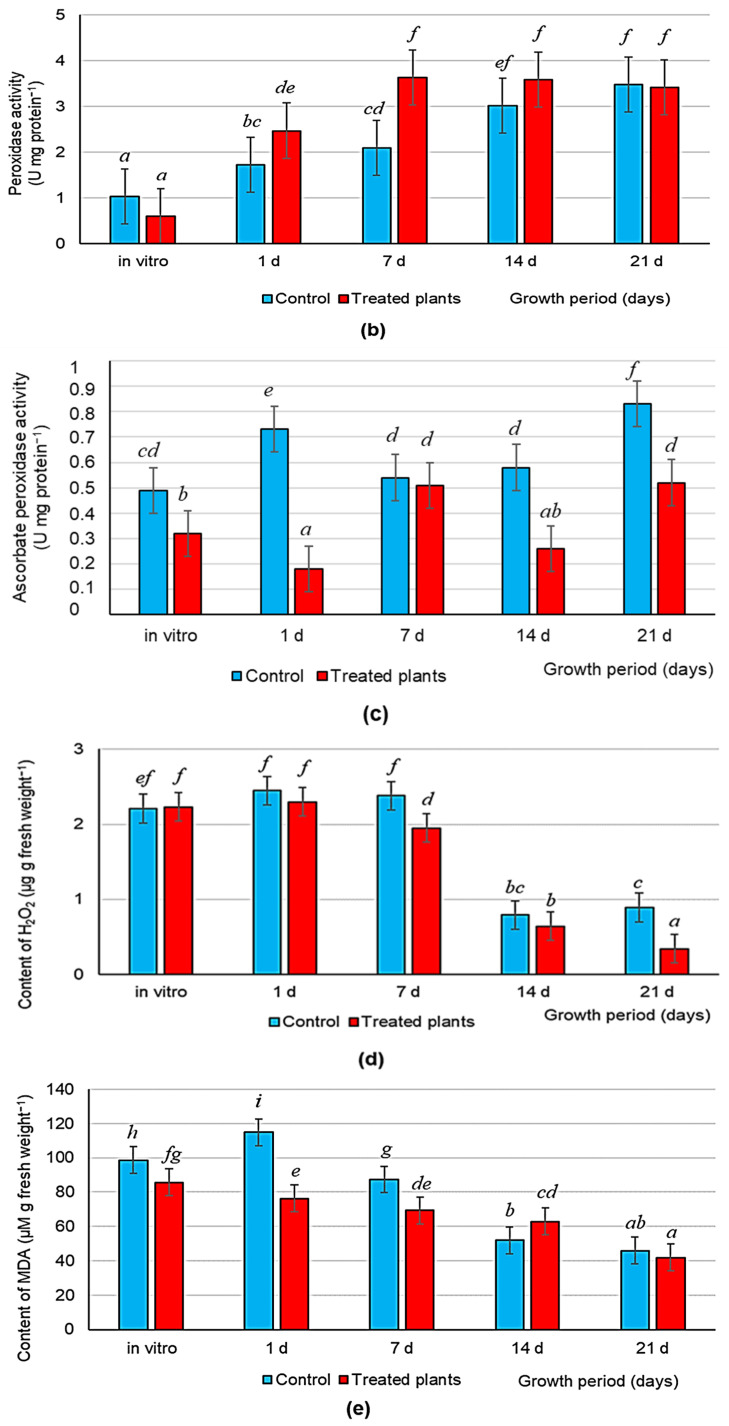
Activity of the pro/antioxidant system in potato plants non-inoculated (control) and inoculated with *A. baldaniorum* Sp245 and *O. cytisi* IPA7.2 (treated plants) in vitro and on days 1, 7, 14, and 21 of aeroponic growth: (**a**) catalase activity; (**b**) peroxidase activity; (**c**) ascorbate peroxidase activity; (**d**) content of hydrogen peroxide; (**e**) content of MDA. Data were processed by one-way and two-way ANOVA (factor A: type of experiment (control and treatment); factor B: growth period (days)). The bars represent LSD for experience options. Latin letters indicate differences between treatments according to the results of the Duncan’s test at *p* ≤ 0.05. *n* = 12.

The activity of the antioxidant enzymes catalase and peroxidase (Figure 5a,b) in leaves did not differ between treated and control plants in vitro. The content of hydrogen peroxide (Figure 5d) was similar in both treated and control plants. In the inoculated plants, the content of MDA (Figure 5e) was decreased by 13.2%, and the activity of ascorbate peroxidase (Figure 5c) was lowered by 34.7%, as compared with the controls.

### 3.2. Plant–Bacterial Associations Ex Vitro

In aeroponics, 100% of the microplants survived. All plants began to form new roots by day 3 of adaptation. New leaves were formed a week later, and the area of existing leaves increased substantially. After 3 weeks of growth (Table 3), the inoculated plants developed significantly greater shoot lengths (+38.9%), fresh and dry weights (+26 and 33.3%, respectively), leaf numbers (+17.8%), and leaf areas per plant (+27.9%) (Table 3). No significant differences were found in root growth between control and treated plants.

The number and size of leaf stomata increased in both control and experimental treatments. In the inoculated plants, the stomata were much smaller than in the control plants, but the stomatal number per leaf area was greater and the stomatal guard cells were more tightly closed than they were in the controls (Table 2 and Figure 2).

Ex vitro, the content of chlorophylls *a* and *b* and of carotenoids per unit leaf weight was higher than it was in vitro, but no differences were found between experimental and control treatments except for chlorophyll *b* at 14 days (Figure 3).

Plant growth in aeroponics was accompanied by a sharp decrease in the number of inoculated bacteria on the roots (to as low as 10^5^ cells per cm of root). The bacterial number then remained at this level for up to 21 days of plant growth inclusive (Figure 4).

On days 1 and 7 of plant adaptation ex vitro, catalase and peroxidase activities in the leaves of inoculated plants were 1.5-fold higher than they were in the controls. On days 14 and 21 of plant growth, peroxidase activity had the same values in both control and experimental treatments (Figure 5a,b).

By contrast, higher ascorbate peroxidase activity was observed in the leaves of control plants throughout their growth ex vitro (Figure 5c). The leaf content of hydrogen peroxide, the main ROS component, was high when the plants started aeroponic growth and gradually decreased in both control and experimental treatments by days 14 and 21 of growth. Of note, the bacteria contributed to a more intense reduction in the leaf content of hydrogen peroxide (Figure 5d). In turn, inoculation contributed to a decrease in the leaf content of MDA on days 1 and 7 of plant growth. By day 21, the MDA content had gradually reached the control value (Figure 5e). Thus, on day one after the plants had been transferred to the aeroponic system, the leaf MDA content of the inoculated plants did not increase, as compared with the controls. This suggests that bacterization contributes to reducing oxidative stress and faster adaptation of plants to ex vitro conditions.

Yield analysis (Table 4) showed that the minitubers did not differ in size or weight between control and treated plants. This was unsurprising, because minitubers were harvested as they reached their standard size. The yield of minitubers per plant in the experimental treatment increased by 11%, as compared with the control treatment. By contrast, the number of minitubers per plant in the experimental treatment was greater than the control value by 30%.

## 4. Discussion

Because plants lead a sessile life, they are often exposed to various environmental factors. At the same time, oxidative stress develops in plants, which disrupts the normal functioning of cells [33,34]. To cope with the oxidative stress caused by adverse conditions, plants have an antioxidant system that includes both enzymatic (catalase, superoxide dismutase, ascorbate peroxidase, etc.) and non-enzymatic (proline, cysteine, glutathione and ascorbic acid) components. Antioxidants maintain a balance between the formation of reactive oxygen species (ROS) and their destruction, thereby preventing damage to various biomacromolecules and ultimately preventing the death of plants [35]. Numerous studies have shown that PGPR regulate the antioxidant defense system of plants, contributing to plant resistance to various kinds of stress [36,37,38].

Ex vitro conditions (in particular, the aeroponic system) may be regarded as a kind of abiotic stress for microplants, which leads to an imbalance in the synthesis and removal of ROS in different cellular compartments. It is important to understand the mechanisms by which bacteria mitigate the effects of stressors on the host plant under ex vitro conditions.

In this study, potato inoculation in vitro with the PGPR *A. baldaniorum* Sp245 and *O. cytisi* IPA7.2 increased catalase and peroxidase activities in the leaves more intensely after potato microclones had been transferred to aeroponics than it did in the control condition (Figure 5a,b). This process was accompanied by a decrease in the content of two major indicators of oxidative stress in plants: hydrogen peroxide (Figure 5d), an important ROS, and MDA (Figure 5e), the final product of lipid peroxidation. By contrast, the bacterization reduced ascorbate peroxidase activity in plants both in vitro and ex vitro. This is probably because the high catalase and peroxidase activities sharply decrease the content of hydrogen peroxide at the initial stages of plant growth ex vitro and because the bacteria reduce the activity of other enzymes directed to the detoxification of peroxide, thereby maintaining the redox balance of cellular homeostasis. It is also possible that the content of ascorbic acid, the main substrate of ascorbate peroxidase in plants, was lowered in the experimental treatments owing to bacterial activity. As a result, inoculation contributed to the regulation of the plant pro/antioxidant system, leading to better and faster survival of plants ex vitro. This is evidenced by the higher values for the morphological and physiological variables of the inoculated plants, as compared with the control values (Table 3). Earlier, we investigated the effect of osmotic stress on potato and found that the increase in catalase activity in the leaves of stressed, inoculated plants led to a decrease in the leaf content of MDA and to a better and more rapid recovery of experimental plants undergoing post-stress repair [39]. Ouhaddou et al. [40] showed that a consortium of *Bacillus* PGPR and arbuscular mycorrhizal fungi improved salt tolerance in lettuce, decreasing the content of malondialdehyde and hydrogen peroxide and increasing leaf peroxidase activity and dry plant biomass. Similarly, Bandeppa et al. [41] found that the inoculation of mustard plants with *Bacillus* rhizobacteria increased catalase activity and decreased the leaf MDA content, contributing to a better and faster recovery of osmotically stressed experimental plants. Other investigators have shown that *Bacillus* bacteria contribute to the resistance of pea to salt stress in the greenhouse by increasing the activity of superoxide dismutase, catalase, and ascorbate peroxidase and ultimately decreasing oxidative stress [42]. Inoculation of common bean (*Phaseolus vulgaris* L.) seeds with *Bacillus* endophytes promotes plant growth in both saline and non-saline environments. The bacteria reduce the leaf content of MDA, thereby neutralizing oxidative damage to plant cells [43].

Monja-Mio et al. [44] showed that the functionality of stomata during plant adaptation depends on the quality of their development in vitro. In this study, when plants were transferred from in vitro to ex vitro conditions, the leaf stomata underwent substantial changes. The decrease in air humidity during transfer from in vitro to ex vitro conditions led to an increase in the number and size of stomata. Under aeroponic conditions, the effect of the rhizobacteria led to the appearance of more stomata of smaller size and with a smaller pore between the guard cells. This indicates their better functionality, that is, the ability to regulate transpiration and gas exchange. This is in harmony with the data of Khai et al. [45], who observed an increase in the adaptation potential of gerbera, with similar findings for stomata owing to the effect of selenium nanoparticles. The obtained data show that the bacterization of plants lead to a better adaptation of stomata and leaves of plants to stressful conditions of reduced air humidity in ex vitro conditions.

Shoot growth was promoted both in vitro and ex vitro. Under the effect of the bacteria, root growth was promoted in vitro, as found earlier [6,7]. Under aeroponic conditions, no significant differences between the roots of the control and treated plants were found on day 21. Apparently, this is because roots are formed de novo and their growth at this stage is less dependent on inoculants.

Ultimately, inoculation of potato microplants with rhizobacteria not only accelerated their adaptation but also increased plant productivity. The increase in productivity was also observed previously [9] for potato plants inoculated singly with *A. baldaniorum* Sp245 and grown in aeroponics.

Despite the fact that *A. baldaniorum* Sp245 is not a native species for potato and has been isolated from wheat [19], the plant-growth-promoting activity of *Azosprillum*, including this strain, is well known for various plant species, including potato [4,46]. *O. cytisi* IPA7.2 was isolated from the roots of the potato cultivar Nevsky [5], and from the experimental data it can be seen that in vitro there are a greater number of cells of this strain in plants. However, this is due to the advantage of *O. cytisi* IPA7.2’s ability to use sucrose of the nutrient medium [5]. Under aeroponics conditions, the content of both strains in plant roots is leveled, which indicates the absence of competitive relations between the strains, which confirms the results we obtained earlier [7].

As shown by our past studies on the effect of bacterization by the same strains of *A. baldaniorum* Sp245 and *O. cytisi* IPA7.2 when grown in soil, the effect of the strains can be manifested both individually and in a consortium [7]. The effect of single *A. baldaniorum* Sp245 was better manifested at the stage of plant cultivation in vitro, and the consortium of the strains had a greater effect after the transfer of plants to the soil. At the same time, from the point of view of increasing the stability of the plant–microbial association, inoculation of plants by a consortium of strains is preferable [47]. Data on the growth-stimulating activity of single *A. baldaniorum* Sp245 in aeroponics coincide with the established effect of co-inoculation by two strains [9]. In contrast to our previous studies, this work establishes mechanisms for increasing the growth activity and productivity of plants through the regulation of the pro/antioxidant activity of the system by rhizobacteria.

## 5. Conclusions

This is the first study to have found how the PGPR *A. baldaniorum* Sp245 and *O. cytisi* IPA7.2 function in the antioxidant protection of potato microclones. This function consists in regulation of the activity of antioxidant enzymes (peroxidase, ascorbate peroxidase, and catalase), which leads to a decrease in the leaf content of MDA and hydrogen peroxide during plant adaptation to growth in aeroponics (ex vitro) and reduces oxidative damage. As a result, the inoculated microplants adapt faster, grow faster than do the non-inoculated controls, and produce more minitubers. Our findings would help to improve the efficacy of innovative agricultural biotechnologies aimed at the transition to highly productive and environmentally friendly potato seed breeding.

## Figures and Tables

**Table 1:** Morphometric variables of potato microclones non-inoculated (control) and inoculated with *A. baldaniorum* Sp245 and *O. cytisi* IPA7.2 (treated plants) on day 30 of in vitro cultivation.

Treatment	Root Length (mm)	Root Number	Shoot Length (mm)	Node Number	Fresh Weight		Dry Weight	
					Shoots (mg)	Roots (mg)	Shoots (mg)	Roots (mg)
Control	59.00 b	9.91 a	78.57 a	8.82	241.87 a	204.67 a	31.53 a	14.40 a
Treated plants	52.11 a	10.33 b	84.38 b	9.02	311.93 b	248.93 b	45.40 b	25.93 b

Data were processed by one-way ANOVA. Latin letters indicate differences between treatments according to the results of the Duncan’s test at *p* ≤ 0.05. *n* = 10.

**Table 2:** Number and size of stomata in potato microclones non-inoculated (control) and inoculated with *A. baldaniorum* Sp245 and *O. cytisi* IPA7.2 (treated plants) in vitro and on days 10 and 21 of aeroponic growth.

Treatment	Stomatal Number per 0.1 mm^2^			Stomatal Area (µm^2^)			Stomatal Pore Width (µm)		
	In Vitro	Day 10	Day 21	In Vitro	Day 10	Day 21	In Vitro	Day 10	Day 21
Control	8.67	15.33	22.33 a	277.11	648.86 b	629.67 b	3.35	4.64	3.83 b
Treated plants	10.00	14.33	29.67 b	267.31	497.61 a	466.93 a	3.21	4.09	2.27 a

Data were processed by one-way ANOVA. Latin letters indicate differences between treatments according to the results of the Duncan’s test at *p* ≤ 0.05. For stomatal number, *n* = 10; for stomatal size, *n* = 30.

**Table 3:** Morphometric variables of potato plants non-inoculated (control) and inoculated with *A. baldaniorum* Sp245 and *O. cytisi* IPA7.2 (treated plants) on day 21 of aeroponic growth.

Treatment	Root Length (cm)	Shoot Length (cm)	Leaf Number	Average Leaf Area (cm^2^)	Leaf Area per Plant (cm^2^)	Fresh Weight		Dry Weight	
						Shoots (g)	Roots (g)	Shoots (g)	Roots (g)
Control	36.00	9.65 a	9.00 a	53.74	442.13 a	4.04 a	1.37	0.39 a	0.08
Treated plants	33.70	13.40 b	10.60 b	53.23	565.32 b	5.09 b	1.36	0.52 b	0.05

Data were processed by one-way ANOVA. Latin letters indicate differences between treatments according to the results of the Duncan’s test at *p* ≤ 0.05. *n* = 10.

**Table 4:** Number and size of minitubers in potato plants non-inoculated (control) and inoculated with *A. baldaniorum* Sp245 and *O. cytisi* IPA7.2 (treated plants).

Treatment	Number of Minitubers per Plant	Average Minituber Weight (g)	Average Minituber Diameter (mm)	Weight of Tubers per Plant (g)
Control	10.1 a	20.9	38.5	228 a
Treated plants	13.2 b	17.8	35.7	254 b

Data were processed by one-way ANOVA. Latin letters indicate differences between treatments according to the results of the Duncan’s test at *p* ≤ 0.05.

## Data Availability

The data presented in this study are available on demand from the first author.
